# Experiences and Perceptions of Ophthalmic Simulation-Based Surgical Education in Sub-Saharan Africa

**DOI:** 10.1016/j.jsurg.2021.04.005

**Published:** 2021

**Authors:** Roxanne Annoh, Lena Morgon Banks, Stephen Gichuhi, John Buchan, William Makupa, Juliet Otiti, Agrippa Mukome, Simon Arunga, Matthew J. Burton, William H. Dean

**Affiliations:** ⁎ International Centre for Eye Health, Department of Clinical Research, London School of Hygiene and Tropical Medicine, London, United Kingdom; † International Centre for Evidence in Disability, Department of Clinical Research, London School of Hygiene & Tropical Medicine, London, United Kingdom; ‡ Department of Ophthalmology, University of Nairobi, Kenya; § Kilimanjaro Christian Medical Centre, Moshi, Tanzania; ‖ Department of Ophthalmology, School of Medicine, Makerere University, Kampala, Uganda; ¶ Department of Ophthalmology, Parirenyatwa Hospitals, University of Zimbabwe, Harare; # Mbarara University & Referral Hospital Eye Centre, Mbarara University of Science and Technology, Mbarara, Uganda; ⁎⁎⁎ National Institute for Health Research Biomedical Research Centre for Ophthalmology at Moorfields Eye Hospital NHS Foundation Trust and UCL Institute of Ophthalmology, United Kingdom; ‡‡ Division of Ophthalmology, University of Cape Town, South Africa

**Keywords:** ophthalmology, training, africa, simulation, education, Practice-Based Learning and Improvement, Interpersonal and Communication Skills, Patient Care

## Abstract

**BACKGROUND:**

Simulation-based surgical education (SBSE) can positively impact trainee surgical competence. However, a detailed qualitative study of the role of simulation in ophthalmic surgical education has not previously been conducted.

**OBJECTIVE:**

To explore the experiences of trainee ophthalmologists and ophthalmic surgeon educators’ use of simulation, and the perceived challenges in surgical training.

**METHODS:**

A multi-center, multi-country qualitative study was conducted between October 2017 and August 2020. Trainee ophthalmologists from six training centers in sub-Saharan Africa (SSA) (in Kenya, Uganda, Tanzania, Zimbabwe and South Africa) participated in semi-structured interviews, before and after an intense simulation training course in intraocular surgery. Semi-structured interviews were also conducted with experienced ophthalmic surgeon educators. Interviews were anonymized, recorded, transcribed and coded. An inductive, bottom-up, constant comparative method was used for thematic analysis.

**RESULTS:**

Twenty-seven trainee ophthalmologists and 12 ophthalmic surgeon educators were included in the study and interviewed. The benefits and challenges of conventional surgical teaching, attributes of surgical educators, value of simulation in training and barriers to implementing ophthalmic surgical simulation were identified as major themes. Almost all trainees and trainers reported patient safety, a calm environment, the possibility of repetitive practice, and facilitation of reflective learning as beneficial aspects of ophthalmic SBSE. Perceived barriers in surgical training included a lack of surgical cases, poor supervision and limited simulation facilities.

**CONCLUSIONS:**

Simulation is perceived as an important and valuable model for education amongst trainees and ophthalmic surgeon educators in SSA. Advocating for the expansion and integration of educationally robust simulation surgical skills centers may improve the delivery of ophthalmic surgical education throughout SSA.

## INTRODUCTION

The 46 countries of sub-Saharan Africa (SSA) are home to 12% of the global population, and 10% of the global burden of visual impairment.^1^^,^^2^ Of the more than 230,000 ophthalmologists worldwide, there are only 2.5 ophthalmologists per million population in SSA.^3^ This compares to 27,000 ophthalmologists for the 323 million people of the United States, a ratio of 83.6 per million; and a global mean of 31.7.^4^^,^^5^

There are 57 training institutions for ophthalmologists in 11 Anglophone, 11 Francophone and two Lusophone countries in SSA.^6^ Setting up training institutions is challenging due to large unmet needs for clinical service provision, limited availability of teaching faculty, time, financial and other resource constraints. Furthermore, the demand for services is often far less than the population need, with relatively low numbers of patients affording and seeking care. At the advent of “VISION 2020: The Right to Sight” in 1999, initial priority actions within SSA included the “development of collaborative regional training programs for ophthalmologists to improve the quality of education and to increase the number of trainees”.^7^

There is increasing evidence of the benefits of simulation-based surgical education (SBSE), however only recently has more comprehensive randomized-controlled trial (RCT) level evidence been available for ophthalmic surgery. The OLIMPICS and GLASS trials conducted by Dean et al. between 2017 and 2019 examined the efficacy of intensive, high-fidelity surgical simulation training on cataract and glaucoma surgical competency in residents based in Eastern, Central and Southern Africa.^8^^,^^9^ These trials demonstrated the superiority of SBSE in improving surgical competency compared to conventional training alone. Yet, whilst these results are promising, further research is needed to explore the acceptability of this newer educational method in ophthalmology, along with the perceived barriers to its implementation in sub-Saharan training institutions. We therefore conducted a qualitative study to investigate the subjective perspectives of ophthalmology trainees and experienced ophthalmic surgeon educators towards the delivery of conventional ophthalmic surgical training and SBSE in Western, Eastern, Central and Southern Africa.

## METHODS

### Study Design

We conducted a multi-center, multi-country qualitative study between December 2017 and August 2020. An interpretivist, epistemological study design was used to obtain an understanding of trainee and ophthalmic surgeon educators’ perspectives of conventional and simulation approaches in surgical education.

### Setting & Participants

The GLAucoma Simulated Surgery Trial (GLASS) was a randomized controlled trial evaluating the efficacy of intensive glaucoma SBSE on surgical competency for trainee ophthalmologists in Kenya, South Africa, Tanzania, Uganda and Zimbabwe. The trial was conducted between October 2017 and July 2019 (trial protocol available as online annex). ^9^^,^^10^ The details of the intensive glaucoma SBSE curriculum can be found in Appendix A. Trainees participating in the trial (from the intervention and control arms) were identified using purposeful sampling. A total of seventeen trainees were interviewed at baseline assessment and 10 different trainees at the time of primary intervention. A purposive sampling strategy was used to maximize heterogeneity by location (across the six post-graduate SSA-based ophthalmology training institutions), varying levels of exposure to intraocular surgical teaching, and ensure representation from each trial arm. The duration of postgraduate ophthalmology training, on which trainees were resident, ranged between 3 to 5 years.^6^

Ophthalmic surgeon educators, with experience of conducting ophthalmic surgical training in SSA, were invited for semi-structured interviews via email correspondence between July and August 2020. Educators were identified using purposeful and snowball sampling, to capture perspectives from trainers based in different regions within SSA (Central, East, West & Southern Africa) and with experience of using both conventional ophthalmic surgical training and ophthalmic SBSE training methods.

### Data Collection

Two semi-structured topic interview guides were developed by experienced trainer ophthalmologists and experts in qualitative research methods (Appendices B & C). Key topics to be explored included attitudes towards conventional training and ophthalmic SBSE, and perceived barriers to its delivery. Interview guides were revised and adapted following feedback from pilot interviews. Semi-structured interviews with trainee ophthalmologists took place face-to-face at a university hospital in Cape Town, South Africa and those with ophthalmic surgeon educators took place remotely, using the encrypted online conferencing software Zoom (Zoom Video Communications, Inc. version 5.0.4). All interviews were conducted, recorded, anonymised and transcribed in English by researchers (RA & WHD), both of whom have received training in qualitative research methods.

Reflexivity was upheld throughout the research process.^11^ WHD, an ophthalmic surgeon educator and the principal investigator of the GLASS trial, conducted all semi-structured interviews with trainee ophthalmologists during the trial period. WHD was also responsible for delivery of SBSE to all participants of the GLASS trial. Interviews with trainee ophthalmologists took place prior to SBSE training and following exposure to SBSE training. RA, a UK-based senior resident ophthalmologist, conducted all interviews with ophthalmic surgeon educators remotely. There was no prior relationship between the interviewers and study participants. The influence of the researchers’ background on knowledge generation was considered during the study design process.

### Data Analysis

Recorded transcripts from the interviews were checked repeatedly for consistency and deconstructed using semantic thematic analysis as described by Braun and Clarke. ^12^ Coding was initiated by RA, using an inductive, bottom-up approach. Initial codes were generated, with expansion and modification of these codes on further analysis. Codes were organized into themes using thematic map analysis using NVivo software version 12.4.0 for Mac (QRS International, Cheshire, UK). Themes were reviewed by RA and WHD, using the constant comparative method and triangulation, to compare the views of trainees and ophthalmic surgeon educators.

### Ethics

This study was approved by the Research and Ethics Committee of the London School of Hygiene & Tropical Medicine (references: 11795 & 21608). All participants were provided with information about the nature of the survey and provided informed consent prior to participation.

## RESULTS

### Respondent Characteristics

A total of 27 out of 49 (55.1%) trainee ophthalmologists from the GLASS trial were selected through purposeful sampling and invited to participate in semi-structured interviews. The mean time in post-graduate ophthalmology residency for trainees was 2.6 years (standard deviation (SD) 0.6) for those interviewed before training and 2.5 years (SD 0.7) after simulation training. Eleven (64.7%) of the trainees interviewed were female (Table 1). Twenty-six ophthalmic surgeon educators were invited to participate. Fifteen (57.7%) educators responded and 12 (46.2%) took part in the interviews. The mean age of educators was 50.3 years (SD 8.5), with 6 (50.0%) being female and 7 (58.3%) reporting at least 10 years’ experience in delivering ophthalmic surgical education (Table 2).

**TABLE 1:** Demographic characteristics of ophthalmology trainees. SD = standard deviation

Ophthalmology Trainees (n = 27)		
	Pre-training (n = 17)	During simulation training (n = 10)
Age in years (mean ± SD)	34.2 ± 2.6	34.7 ± 3.3
Female gender, n (%)	11 (65)	5 (50)
Time in residency in years (mean ±SD)	2.6 ± 0.6	2.5 ± 0.7

**Geographical location of SSA training institution**		
Kilimanjaro Christian Medical Centre, Tanzania	4 (23.5)	2 (20.0)
Mbarara University & Referral Hospital Eye Centre, Uganda	4 (23.5)	1 (10.0)
Makere University, Uganda	2 (11.8)	0 (0)
University of Nairobi, Kenya	5 (29.4)	4 (40.0)
University of Zimbabwe, Zimbabwe	2 (11.8)	2 (20.0)
Groote Schuur Hospital & Red Cross Children's Hospital, South Africa	0 (0)	1 (10.0)

**TABLE 2:** Demographic characteristics of ophthalmic surgeon educators

Ophthalmic surgeon educators (n = 12)	
Age in years (mean ± SD)	50.3 ± 8.5
Female gender, n (%)	6 (50.0)
Availability of dry or wet lab facilities in training institution, n (%)	8 (66.7)
Previous participation in the COECSA-RCOphth TTT course n (%)	6 (50.0)
Experience as a surgical trainer in years (n (%))	
3-5 years	1 (8.3)
6-10 years	4 (33.3)
10+ years	7 (58.3)
Geographical location of educators’ past residency programme (n %)	
Outside of SSA	3 (25.0)
East Africa	3 (25.0)
Southern Africa	6 (50.0)
Geographical location of educators’ current training institution (n %)	
West Africa	1 (8.3)
Central Africa	5 (45.5)
East Africa	1 (8.3)
Southern Africa	5 (45.5)

COECSA = College of Ophthalmology of Eastern, Central and Southern Africa; RCOphth = Royal College of Ophthalmologists; TTT = Training the Trainer

### Thematic Analysis

Five over-arching themes and sub-themes were identified (Table 3). The following sections summarise each theme with verbatim quotations used for illustration.

**TABLE 3:** The five overarching themes and associated sub-themes

Main Theme	Sub-themes
Benefits of Conventional Training	Tackling the magnitude of disease burden
	Theoretical-based learning and observation
	Trainee motivation to provide the best patient outcomes
	Collaborative surgical learning
Ethical Challenges in Conventional Training	Unstructured training techniques
	Apprenticeship teaching model
	Risk to patients
	Stress & anxiety relating to live surgical teaching
Attributes of Ophthalmic Surgeons & Surgeon Educators	Positive attributes of surgeon educators
	Negative attributes of surgeon educators and surgeons in training
Value of Simulation in Ophthalmic Surgical Education	Stress reduction
	Technical skill development
	Confidence building
	Repetitive practice
	Reflective learning
Barriers to Implementation of Ophthalmic Simulation-Based Surgical Education	CostInadequate supervisionLack of equipment or facilitiesResistance to changeSustainability

### Benefits of Conventional Ophthalmic Surgical Training

Trainees felt most motivated and confident in their surgical skills when their surgical cases resulted in satisfactory patient outcomes. Many perceived this as confirmation of skill acquisition and safe surgical practice. For most trainees, restoring vision was at the root of pursuing a career in ophthalmology:

“Patients are the ones that motivate me. You get to perform cataract surgery and then the patient gets to see the next morning…that's what motivates me most, the outcome of the patient.” (Trainee in Tanzania)

Many educators felt that witnessing the growth of trainees in surgical competency (from novice to proficient) was a rewarding factor in delivering surgical education. One educator in Southern Africa felt duty bound to train as a means of tackling the magnitude of eye disease burden in the region:

“I believe that if you are honest with yourself, you have to do something, to make the world a better place. I believe that if you can train somebody else, you actually exponentially increase the possibility that people will be helped.”

Educators with negative experiences during their own surgical training preferred a more stepwise approach to training. This approach consisted of training using theoretical-based learning, observation and SBSE in the early stages of residency, followed by live surgical teaching for advanced skill development. However, some trainees perceived live surgery as integral for technical skill acquisition and for improving both surgical competence and self-confidence. A trainee in Uganda provided their views on the best technique for learning ophthalmic surgery:

“The most important way to learn surgery? You need to be exposed to many patients. You can only improve by doing that surgery. If you are doing this you are improving, if you are not, then you do not improve.”

Collaborative surgical learning, through the exchange of surgical tips with other surgeons and observation of colleagues’ surgical practice, was favored by many educators. Collaborative exchange with trainees was also considered as an important learning opportunity for progression of educators’ own skill development:

“Sometimes young surgeons are also extremely inspiring, because they think outside the box and they think of new ideas…I like them asking questions because then you can improve in that way.” (Educator in Southern Africa)

### Ethical Challenges in Conventional Ophthalmic Surgical Training

Good quality ophthalmic surgical training was perceived as training that is patient safe, comprehensive and structured. Patient safety, in particular, was considered paramount in the delivery of surgical training by both trainees and educators. Some trainees expressed unease about developing their surgical skills during live surgery on complex patient cases, particularly in the absence of supervision, for fear of causing harm due to inexperience. Similarly, educators found live surgical training with junior trainees stressful and overwhelming due to the high risk of complications. One educator in Southern Africa with experience in glaucoma surgical teaching stated:

“I don't like training difficult procedures like…trabeculectomies, which can have nasty outcomes...because it's just not fair to be doing a procedure that you're not familiar with for your first time on a live patient...you can't really afford to go wrong.”

The apprenticeship model was the most common method observed in ophthalmic surgical training by trainees and educators. Stress and anxiety were reported highly with those surgically trained with this model.

“The strategy for teaching was really just frying pan style, meaning just like, throw you in. We don't utilize that model of training now… because I think that's a bad way to learn. I mean, I did okay… but it was not great for me or the patients.” (Educator in Central Africa)

### Attributes of Ophthalmic Surgeons & Surgeon Educators

Educators perceived good ophthalmic surgeons as having a broad surgical experience, flexibility with surgical techniques, respect for ocular tissue, and being safe and insightful. When reflecting on past experiences of surgical training, educators considered a calm demeanor, constructive feedback and prioritizing patient safety as the most important qualities of a surgical educator. One educator in Central Africa suggested:

“You need three things to be a good eye surgeon. You need the eyes of an eagle; you must see well. Then you need the heart of a lion because you must be courageous, and you need the hands of a princess.”

**FIGURE 1:**
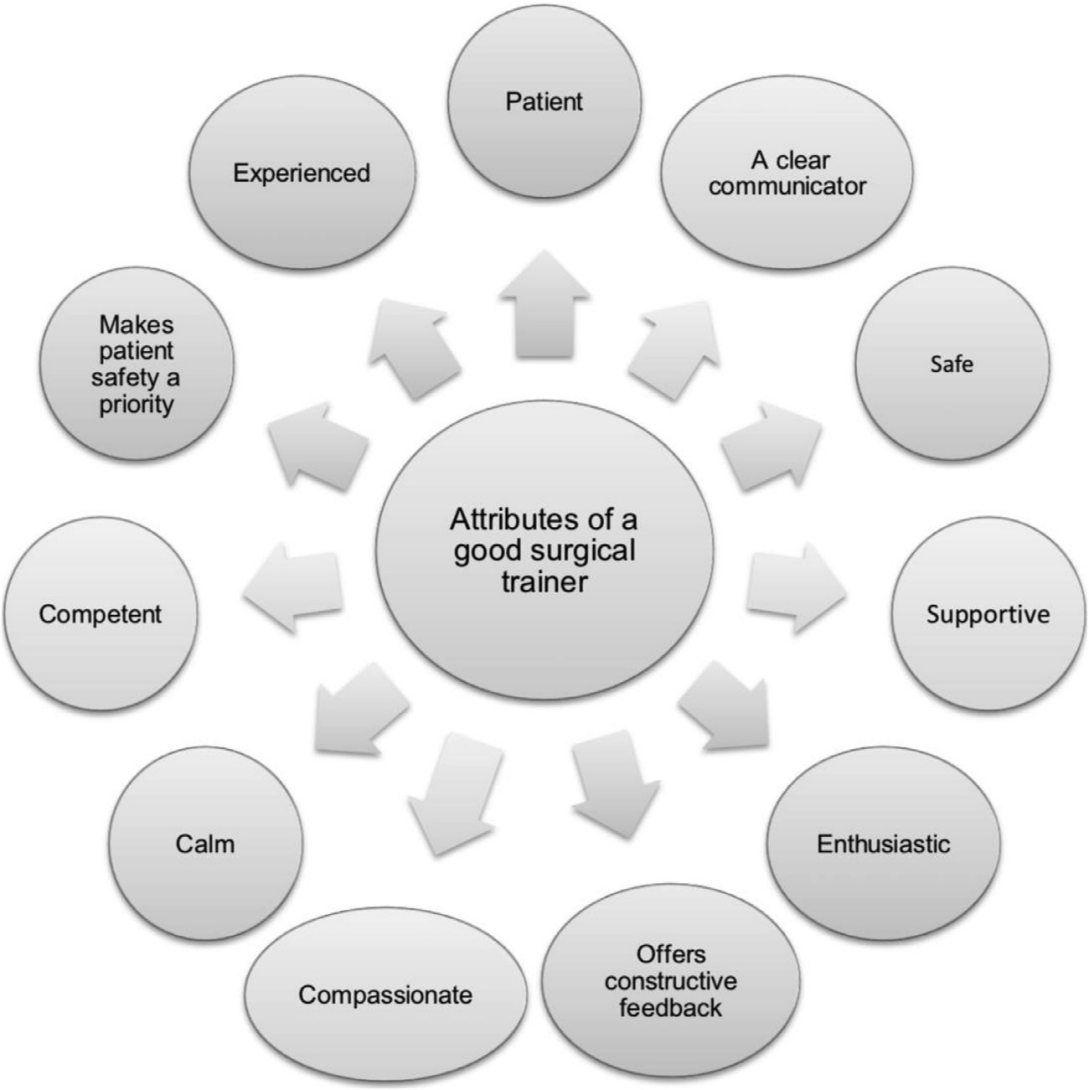
Perceived attributes of a good ophthalmic surgical trainer. A summary of the most common attributes mentioned by educators and trainees.

Patience, good communication, supervision and enthusiasm were rated highly by trainees as positive attributes of a good surgical trainer (see Fig. 1). One trainee in South Africa associated supervision with better rates of skill acquisition:

“I had one consultant teaching me and he was with me in theatre the whole time and by that way, I learnt a lot from him.”

**FIGURE 2:**
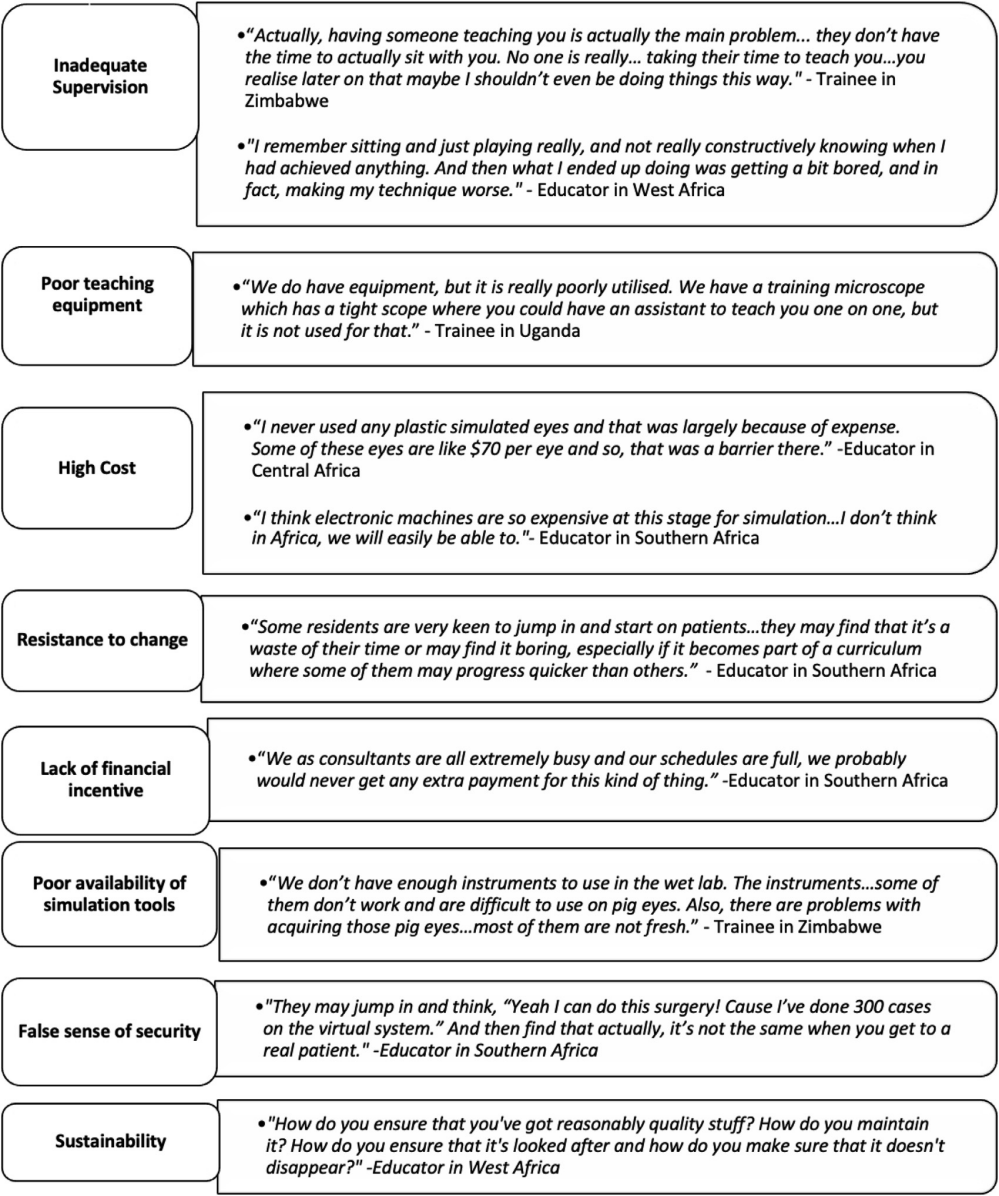
Perceived barriers to implementation of simulation-based ophthalmic surgical education.

Overconfidence, poor decision-making and irrationality in junior surgeons were viewed negatively by educators, where some associated this with unsafe surgical practice leading to poor outcomes and patient harm. For trainees, behaviors such as taking over of a case, poor management of complications, inadequate explanation of surgical steps and performing surgery in a time- pressurized environment were reported as barriers to surgical learning. Some educators and trainees described their own experiences of poor surgical training, resulting in anxiety, despair and guilt:

“Some trainers would pay no attention at all and were really useless...or they'd be so nervous themselves that they transmitted that to you” (Educator in East Africa)

“They made emphasis on the complication and stop you from going through the procedure.... you feel bad.” (Trainee in Zimbabwe)

Complications were perceived as a stumbling block in surgical learning. Receiving little constructive feedback or opportunity to reflect on surgical errors were a source of frustration for trainees, who also attributed this as a barrier to confidence building. Engagement in reflective practice, in both positive and negative aspects of surgical training, was seen as essential for maintaining good working relationships between trainees and educators.

### Value of Simulation in Ophthalmic Surgical Education

SBSE was widely accepted by both trainees and surgeon educators. Reasons for this related to patient safety concerns during live surgical teaching and apparent faster acquisition of surgical skills compared to traditional surgical teaching. For one educator in East Africa, simulation helped to reduce stress at the time of operating:

“You can start, you can pace yourself and you can actually get proficient on a model before you even get onto a real patient. I think it's less stressful on you, less stressful on the patients, because the patients are being operated on by somebody who is at a much higher level of proficiency.”

Yet, not all educators considered simulation as essential for skill acquisition:

“I didn't go through wet lab training...we learnt on live patients. Even phaco...even corneal surgery... I haven't done any wet lab training because we have not had any set up and I'm doing just fine. (Educator in East Africa)”

Repetitive practice and targeted skill development using wet lab facilities were perceived by trainees to improve technical skills such as manual dexterity and eye to hand coordination. Trainees also felt more confident, safer and supported in the simulated environment, especially on receiving constructive feedback by their supervisors:

“You get to make mistakes without facing the consequences of the mistakes. So, you are given a time to make as many mistakes as you can and to learn from those mistakes without the fear, the shouting, the tension, in a relaxed environment. So, it sticks better than learning in a real environment, whereby you face challenges with the surgeons, trainer and you, yourself, and you are not allowed to make many mistakes.” (Trainee in Tanzania)

“It allows you to start getting over that first psychological barrier and you are not worried about complications. So you can be confident when you are going to operate on someone.” (Trainee in Zimbabwe)

### Barriers to Implementation of Ophthalmic Simulation-Based Practice

The perceived barriers to adopting SBSE were multifactorial (Fig. 2). For trainees, the frequency and availability of surgical teaching depended on the suitability and number of cases, the type of surgery and the availability of supervision. Most trainees reported undertaking SBSE completely independently and some felt that more guidance was needed during practice. Both trainers and educators also described poor surgical teaching equipment as a barrier. Resistance to using SBSE by faculty members was another concern described by some educators, owing to inexperience and unavailability of simulation tools and facilities. Equally, many felt that a purely simulation-based surgical curriculum would confer a false sense of security amongst trainees, who may be assessed as competent in simulated surgery but be unable to safely transfer their surgical skills during live surgery.

## DISCUSSION

The aim of this study was to explore the attitudes of trainee ophthalmologists and ophthalmic surgeon educators towards conventional and simulation approaches in ophthalmic surgical training in SSA. Our findings suggest that the burden of ophthalmic disease and visual impairment is the most important driver for improving ophthalmic surgical training across the continent. Yet, trainee dissatisfaction with ophthalmic surgical training is apparent, largely relating to inadequate constructive feedback and time-pressured learning during live surgery. Educators also report similar dissatisfaction with their own surgical learning at the time of their training. This finding is reflected in a recent survey of ophthalmology trainees in SSA which illustrated that although 72% were satisfied overall with their ophthalmology training program, only 48% and 43% were satisfied with cataract and non-cataract surgical training respectively.^13^

Clearly, the culture of the training environment is critical. Trainees’ appreciation of patience, and not anger or impatience, is in line with surgical education throughout the world, but may be more so in SSA. As Thomas Fasokun, Professor of Adult Education at Ile-Ife (Nigeria) concludes: “anxiety is one issue that dominates the participation of African adults in learning. For learning to take place, the level of anxiety in the learner must be minimized. Adults are quick to react unfavorably to unnecessary pressure on them”.^14^ Employing humanist adult-learning teaching methods, such as those that encourage adult learners the freedom of choice, the ability to learn in a non-threatening environment and the opportunity for self-evaluation, is therefore highly recommended. A narrative review of surgical education and adult learning has identified numerous themes, including the importance of patience and tolerance.^15^

It is often a challenge within ophthalmology training institutions, sometimes oversubscribed, for surgical trainers to have enough time and appropriate cases to teach surgery effectively. Furthermore, the Covid-19 pandemic has profoundly impacted surgical specialty training worldwide.^16^^,^^17^ This is due to the suspension of normal (conventional) residency program structures, cancellation of routine (elective) services and face-to-face educational events. Consequently, practical surgical training opportunities have dramatically fallen. Many surgical educators are therefore keen to explore more innovative and effective ways to train efficiently and safely. In this study, collaborative surgical learning and SBSE methods were viewed favourably, largely because of the perception – which is also documented in the literature – that the apprenticeship model is associated with significantly higher rates of adverse patient outcomes.^18^, ^19^, ^20^, ^21^, ^22^, ^23^ This is in line with published findings in Europe where SBSE, in other surgical specialties, is highly regarded by trainees as a positive learning tool for technical skill development and confidence building in the absence of patient harm.^24^^,^^25^ SBSE is evolving and can now be delivered in both face-to-face and in remote settings, using both low-fidelity and high-fidelity models and with appropriate safety measures.^26^ As a result, it is considered as a suitable alternative to conventional training methods that require patient interaction.

However, ophthalmic SBSE in SSA is underutilized and unstructured. Inaccessibility to simulation facilities is evident, with only 8 out of 12 educators interviewed having access to dry or wet lab simulation facilities in their own training institutions. Other factors such as cost, inadequate supervision, resistance to change from conventional teaching methods and a lack of functioning surgical teaching equipment are also perceived as barriers to the formal integration of SBSE into the ophthalmic surgical curriculum. SBSE offers the potential to substantially enhance the quality, speed and safety of surgical skills acquisition.^8^^,^^22^ Yet, for it to work, instruction, supervision, feedback, a curriculum and outcome (surgical competency) measurement are required.^27^ The use of high-fidelity simulation models is not essential for delivery of good quality simulation teaching. In fact, educators in low-and-middle income settings are encouraged to use cheaper, low-fidelity models for training, of which many examples are available.^28^^,^^29^ Thus, a structured, ophthalmology residency curriculum that incorporates stepwise, low-fidelity simulation training and assessment early in training, with safe transition to live surgical teaching presents an excellent opportunity for educators to improve the overall quality of ophthalmic surgical teaching in the region.

### Limitations

The characteristics of the study participants did not appear to significantly influence the perceived experiences reported yet we must recognize that the views were largely from those based in Eastern and Southern Africa. Analysis of the qualitative findings therefore cannot be generalized to other African regions. Similarly, as most educators had access to simulation facilities in their institutions, the views represented may be biased in favor of SBSE. A further study to examine the views of trainers who are inexperienced in SBSE, or with more representation from other African regions may yield different but important results. Triangulation and the constant comparative method were adopted to compare and evaluate perceptions between educators and trainees; however, interviews and thematic analysis were conducted solely by RA and WHD, both of whom have experience in SBSE. Furthermore, as WHD was the primary educator in the GLASS trial, this may have influenced participants’ comments on the subject matter as well as introduced unconscious bias when interpreting results.

## CONCLUSION

To our current knowledge, this is the first known study to provide a comprehensive, qualitative exploration of the attitudes towards conventional ophthalmic surgical training and SBSE amongst ophthalmic educators and trainees in SSA. The high pressure and stressful environment of the operating theatre, the huge burden of ophthalmic disease, and the need for a calm environment to learn and practice surgery arguably strengthen the case for formal ophthalmic SBSE programs in future ophthalmic surgical training. The findings are not only pertinent to ophthalmic surgical training but to wider surgical education and practice. Yet, whilst SBSE is widely accepted, factors such as cost, motivation, practicality and access remain important barriers for local implementation. Advocating for the expansion and integration of educationally robust simulation surgical skills centers may improve the delivery of ophthalmic SBSE throughout SSA. In doing so, one can achieve better standards of ophthalmic surgical training and most importantly, improve the delivery of safe and effective ophthalmic surgical care in SSA.

## ACKNOWLEDGMENTS

The authors would like to acknowledge Dr Shaffa Hameed for her comments on the qualitative study design.

## Figures and Tables

**Table:** 

Pre-Course	• Formal baseline multiple-choice test of knowledge of basic and clinical sciences • Video of procedure (Trabeculectomy) • On-line basic and clinical sciences lectures (anatomy, physiology, epidemiology, surgery)
Course Curriculum	• Video of procedure (Trabeculectomy) • Epidemiology & Burden of Disease group work • Basic microsurgical skills (suturing) practical • Learning theory lecture • Learning & Assessment tools: Sim-OSSCAR^30^ • Screening and pre-operative assessment group work • Surgical procedure: practice of individual steps: ○ Deconstructing surgical technique into constituent steps: Instruction of each step, feedback of performance, and sustained deliberate practice • Simulation surgical trabeculectomy: ○ Performance using artificial model eye, recording of procedure, self-assessment and reflective learning • Complications and how to manage them lecture • Post-operative care and monitoring (audit) of outcomes lecture

**Table:** 

Semi-structured interview questions	
Section 1: Personal Experience with Surgery in Training/Residency	What was your training in intraocular surgery like? (*More specifically, what was your training in trabeculectomy surgery like?*)
	Please tell me what you found most challenging about your training in intraocular surgery? *(How did you cope with these challenges?)*
	Please tell me what you enjoy most about surgery?
	What do you think makes a good intraocular surgeon?
	If you think about the best surgical trainer you've ever worked with, what attributes did they have? *(Are you able to give an example? What made them so good as a trainer?)*
	What do you find most helpful in developing your own surgical skills?
Section 2: Personal Experiences as A Surgical Trainer	What motivates you as a surgical trainer?
	How do you think intraocular surgery is best taught?
	How do you personally train your residents in intraocular surgery? *(If you face any challenges, what are these?)* *(What methods are most useful for teaching trabeculectomy surgery to your residents?)*
Section 3: Experiences & Views on Ophthalmic Surgical Simulation	What is your opinion on using ophthalmic simulation surgical training and education?
	What are your personal experiences of using ophthalmic simulation techniques to train yourself and others? *(If no experience, how do you envision using these methods in your future practice?)* *(If not, what are your reasons for this?)*
	What is the future of surgical training in trabeculectomy and other intraocular surgery?
	What are the challenges (if any) that you foresee with surgical simulation training in intraocular surgery with residents?
Section 4: Views on Ophthalmic Surgical Simulation Courses	If you had the opportunity to run a cataract ophthalmic surgical simulation course in your training institution (or an SSA-based institution), how would you go about this? *(Please think about whether you would use local or external faculty, dry/wet lab materials, face-to-face/online materials and the frequency of courses.)*
	Which residents would you target for this cataract simulation course? *(At what point in training/residency do you think simulation courses are important?)*
	If you perceive any challenges with this, what are these?
	And finally, apart from a course in cataract surgical simulation, what other surgical simulation courses do you think residents would benefit from?

**Table:** 

Semi-structured interview questions
What do training surgeons say are the most important ways to learn surgery?
How do you, or how have you, learnt surgery?
What are the main challenges (in your area) in surgical training?
How do you think surgeons can continually improve their surgical skills?
Think about the best surgical trainer you have worked with. What made them so good?
Think about the worst surgical trainer you have worked with. What made them bad?
What, if any, are the main benefits of simulated ocular surgery training?
Does anything motivate you as a surgeon?
What areas could you use most help within surgical training and why?
